# Letter to the Editor regarding “Brazilian Academy of Paediatric Otorhinolaryngology Task Force – lingual frenulum disorders in childhood – evidence-based recommendations”

**DOI:** 10.1016/j.bjorl.2026.101881

**Published:** 2026-07-31

**Authors:** Juliana Alves de Sousa Caixeta, Vitor Guo Chen, Carolina Sponchiado Miura

**Affiliations:** a Academia Brasileira de Otorrinolaringologia Pediátrica, Brazil; b Universidade Federal de São Paulo, São Paulo, SP, Brazil; c Universidade Estadual de São Paulo (USP-RP), Ribeirão Preto, SP, Brazil

*Dear Editor,*

We thank Martinelli and Berretin-Felix for their interest in our Task Force document. Our review screened more than 300 articles, but the journal limits citations to 75, which constrained the works individually referenced. The original protocol by Martinelli et al. was cited under the same criterion applied to all other instruments reviewed.^1^ The 2016 studies ‒ “Validation of the Lingual Frenulum Protocol for Infants” and “Validity and reliability of the Neonatal Tongue Screening Test” ‒ describe the same population in two journals, which raises a legitimate concern of duplicate publication.^2^,^3^

Regarding the validation process itself, several methodological points justify our position that no available instrument has undergone adequate internal or external validation. First, the NTST validation was retrospective, whereas validation of diagnostic questionnaires should be prospective. Second, no pilot study (typically 15–60 participants) was described. Third, although the population was Brazilian, no formal translation procedure ‒ independent forward translation, reconciliation, and back-translation ‒ was reported. Fourth, internal consistency was not assessed by Cronbach’s alpha or any equivalent measure. Fifth, in the intra-observer analysis, only 36 of 100 patients were reassessed and half had undergone frenotomy between evaluations, introducing intervention bias incompatible with reliability testing. Finally, the inter-observer analysis was performed indirectly through photographs and videos from the primary examiner’s archive, using the Intraclass Correlation Coefficient rather than Cohen’s or Fleiss’s kappa, the standard for categorical agreement.^4^, ^5^, ^6^, ^7^, ^8^, ^9^ These observations were applied uniformly to every instrument reviewed.

Concerning the claim of improper use of the NTST image, the figure was reproduced from Martinelli et al. published in Revista CEFAC under the Creative Commons Attribution licence (CC BY), and was cited as reference 75 of the Task Force document, with the source explicitly indicated beneath the image.^1^^,^^10^^,^^11^ The CC BY licence expressly authorizes copying, redistribution, adaptation, and transformation of the material in any medium, including for commercial purposes, conditional solely upon appropriate attribution ‒ a requirement fully met.^12^ The Brazilian Journal of Otorhinolaryngology adopts the same CC BY 4.0 licence, ensuring full compatibility between source and reproducing publication. The WAME recommendations address general principles of publication ethics and do not override the formal legal framework of Creative Commons licences.^13^ Brazilian copyright law (Law nº 9610/1998, Article 46-III) likewise authorises citation of any work for purposes of study, criticism, or controversy, provided author and source are indicated.^14^ Notably, the same criteria were applied to the Hazelbaker, Kotlow, Coryllos, BTAT, and TABBY instruments, whose figures were reproduced under identical conditions without objection.

Our considerations were of a strictly scientific nature, applied uniformly to all instruments. We reaffirm that the methodological appraisal and the reproduction of the figure complied fully with the prevailing scientific, ethical, and legal standards.

## Data availability statement

The authors declare that all data are available in repository.

## Declaration of competing interest

This manuscript did not receive any kind of fund. The authors declare no conflicts of interest.

The authors exceed seven because it is a task force, that evolves a very huge literature review and requests a debate about the statements. It is important to discuss every theme with specialist in each subject.
